# Inhibition of Nickel Nanoparticles-Induced Toxicity by Epigallocatechin-3-Gallate in JB6 Cells May Be through Down-Regulation of the MAPK Signaling Pathways

**DOI:** 10.1371/journal.pone.0150954

**Published:** 2016-03-04

**Authors:** Yuanliang Gu, Yafei Wang, Qi Zhou, Linda Bowman, Guochuan Mao, Baobo Zou, Jin Xu, Yu Liu, Kui Liu, Jinshun Zhao, Min Ding

**Affiliations:** 1 Department of Preventative Medicine, Zhejiang Provincial Key Laboratory of Pathological and Physiological Technology, Medicine School of Ningbo University, Ningbo, Zhejiang, China; 2 Toxicology and Molecular Biology Branch, Health Effects Laboratory Division, National Institute for Occupational Safety and Health, Morgantown, West Virginia 26505, United States of America; 3 Department of Science Research and Information Management, Zhejiang Provincial Center for Disease Control and Prevention, Hangzhou, Zhejiang, China; Rutgers, the State Univesity of New Jersey, UNITED STATES

## Abstract

With the rapid development in nanotechnology, nickel nanoparticles (Ni NPs) have emerged in the application of nanomedicine in recent years. However, the potential adverse health effects of Ni NPs are unclear. In this study, we examined the inhibition effects of epigallocatechin-3-gallate (EGCG) on the toxicity induced by Ni NPs in mouse epidermal cell line (JB6 cell). MTT assay showed that Ni NPs induced cytotoxicity in a dose-dependent manner while EGCG exerted a certain inhibition on the toxicity. Additionally, EGCG could reduce the apoptotic cell number and the level of reactive oxygen species (ROS) in JB6 cells induced by Ni NPs. Furthermore, we observed that EGCG could down-regulate Ni NPs-induced activator protein-1 (AP-1) and nuclear factor-κB (NF-κB) activation in JB6 cells, which has been shown to play pivotal roles in tumor initiation, promotion and progression. Western blot indicated that EGCG could alleviate the toxicity of Ni NPs through regulating protein changes in MAPK signaling pathways. In summary, our results suggest that careful evaluation on the potential health effects of Ni NPs is necessary before being widely used in the field of nanomedicine. Inhibition of EGCG on Ni NPs-induced cytotoxicity in JB6 cells may be through the MAPK signaling pathways suggesting that EGCG might be useful in preventing the toxicity of Ni NPs.

## Introduction

NPs refer to particles with one dimension that measure 100 nm or less [1]. With the fast development in nanotechnology, Ni NPs are widely used in hydrogen storages, chemical catalysts, ceramic capacitors, sensor and conductive paints, and nanomedicine over the past decade [2]. However, public concerns have been aroused on the adverse effects of Ni NPs to the environment and human health [3]. Skin allergies, lung fibrosis, lung cancer and hepatotoxicity damage are the common adverse health effects of Ni fine particle exposure, which had been demonstrated by both *in vitro* and *in vivo* experiments and limited epidemiological studies [4–6]. Meanwhile, evidence showed that Ni NPs might be more carcinogenic than Ni fine particles [7]. Park *et al* reported that 100 nm Ni particles could induce apoptosis and DNA damage by promoting the production of ROS [8,9]. Zhao *et al* demonstrated that Ni NPs could induce more cell apoptosis than Ni fine particles in JB6 cells at the same dose, and Ni NPs could also significantly up-regulate the protein expression levels of the proto-oncogene *Bcl-2* and anti-apoptotic factor *AKT* [10]. In addition, Pietruska *et al* found that Ni NPs activated the HIF-1α signaling pathway, which could induce cell malignant transformation [11]. Another study *in vivo* showed that the formation of rhabdomyosarcomas was observed in rats through intramuscular injection with Ni NPs at the vertebral column [12]. Although our previous studies had demonstrated that Ni NPs might be more harmful than Ni fine particles [13], the carcinogenic cytotoxicity of Ni NPs and the underlying molecular mechanism are still unclear.

EGCG is a major component of polyphenols in green tea [14,15]. It has inhibitory effects on cell transformation and early cancerization, ROS generation and DNA damage induced by inflammation [16,17]. Previous studies of the nude mouse tumorigenicity assay suggested that EGCG might effectively inhibit the growth of prostate cancer cells *via* intraperitoneal injection [18]. Additionally, Wing *et al* found that EGCG could promote the apoptosis of human liver cancer cells by up-regulating the expression levels of *miR-16* and down-regulating the expression levels of *Bcl-2* [19]. The possible mechanism might be that EGCG could inhibit liver cancer cells proliferation through up-regulation of *P53* expression and activation of Fas/FasL signaling pathways [20]. Available studies also suggested that the potential anti-carcinogenic mechanism of EGCG might involve the MAPK, JAK/STAT, PI3K/AKT, Wnt and Notch signaling pathways [21]. In addition, EGCG might inhibit the tumorigenesis through down-regulation of the activations of protein kinases, transcription factors (AP-1 and NF-κB) and growth factor receptors [21].

Therefore, we attempted to identify the inhibitory effects and the potential molecular mechanism of EGCG on Ni NPs-induced cytotoxicity in this study.

## Materials and Methods

### Materials

Ni NPs (the main components: 99.8% Ni, 0.01% Co and 0.0068% Ca) were purchased from Danyang City Alloy and Steel Refinery Co, LTD (Danyang, Jiangsu, China). EGCG (from green tea, E4143, purity > 95%) and 3-(4,5-dimethylthiazol-2-yl)-2,5-diphenyl tetrazolium bromide (MTT) were purchased from Sigma-Aldrich^®^ (Saint Louis, Missouri, USA). The JB6 cells (a mouse epidermal cell line) were received as a gift from the National Institute of Occupational Safety and Health (Morgantown, West Virginia, USA). The kits for bicinchoninic acid (BCA) protein quantitation and the ROS detection were purchased from Beyotime Institute of Biotechnology (Shanghai, China). The cell cycle kit was purchased from MultiSciences Biotech Co, Ltd. (Hangzhou, Zhejiang, China). The Annexin V-FITC/PI apoptosis detection kit was supplied by Invitrogen Corporation (Carlsbad, California, USA). Mouse-anti-human GAPDH monoclonal antibody was obtained from KangChen Bio-tech Inc. (Shanghai, China). The rabbit-anti-human monoclonal antibodies including p-ERK1/2 (phosphorylated ERK1/2), ERK1/2, p-p38 (phosphorylated p38), p38, p-JNK (phosphorylated JNK) and JNK were obtained from Cell Signaling Technology (Danfoss, Massachusetts, USA). Luciferase assay system and TPA (phorbol-12-myristate-13-acetate) were purchased from Promega Corporation (Madison, Wisconsin, USA). The fluorescent protectant (Flu-G) was supplied by Southern Biotechnology Associates (Birmingham, Alabama, USA). The pre-dyed protein marker was purchased from Fermentas Inc. (Republic of Lithuania). Western Bright^™^ ECL was purchased from Asvansta Inc. (Menlo Park, California, USA). All other materials were obtained from Solarbio^®^ (Bejing, China), Beyotime^®^ (Shanghai, China), Sigma-Aldrich^®^ (St. Louis, Missouri, USA), Molecular Probes^®^ (Eugene, Oregon, USA) and Amresco^®^ (Solon, Ohio, USA), respectively.

### Methods

#### Preparation and physical characteristic detection of Ni NPs

Ni NPs (10 mg) were added into a sterile glass bottle containing sterile culture medium (10 mL), then sealed with joint sealant and sonicated for 30 min in an ultrasonic water bath apparatus. Then, the Ni NPs were distributed evenly in culture medium (1 μg/μL). A scanning electron microscope (SEM) was used to evaluate the physical characteristics of the Ni NPs.

#### Cell culture

The JB6 cells were maintained in 10% bovine calf serum DMEM containing 1% penicillin-streptomycin at standard culture conditions (37°C, 80% humidified air and 5% CO_2_). For all treatments, cells were grown to 80% confluence.

#### Cytotoxicity assay

Cytotoxicity of Ni NPs to JB6 cells and the inhibition effect of EGCG were assessed by the MTT assay. Briefly, the JB6 cells were plated at a density of 10,000 cells/well in a 96-well plate with 100 μL culture medium per well. The cells were maintained at standard culture conditions for 24 h, and then treated with Ni NPs alone or Ni NPs + EGCG (the concentrations of Ni NPs: 0, 2.5, 5, 7.5 and 10 μg/cm^2^; the concentration of EGCG: 10 μM). After 24 h incubation, the culture medium was removed and the wells were washed lightly with sterile PBS, then 20 μL MTT solution (3.5 mg/mL) and 180 μL fresh culture medium were added in each well. The plates were further incubated for 4 h. Next, 150 μL DMSO was added into each well and the two plates were incubated on an incubator shaker for 10 min at 37°C. The optical density (OD) of each well was measured at the wavelength of 492 nm.

#### Detection of cell cycle

A cell cycle kit was used to detect the cell cycle of JB6 cells. Briefly, cells were seeded into two 6-well plates for 24 h, then treated with Ni NPs alone or Ni NPs + EGCG for 24 h. Cells were washed two times with 4°C sterile PBS, and harvested by trypsinization. After centrifugation, cell pellets were resuspended in fresh DMEM medium. PI (propidium iodide) dye was added into the cell suspension and cells were further incubated for 30 min, avoiding light. The cell cycle was monitored by flow cytometry.

#### Detection of apoptosis

An AnnexinV-FITC/PI kit was used to detect cell apoptosis. Briefly, cells were collected, which was similar to the cell cycle detection. Cells were gently added to a flow cytometry tube containing 500 μL Annexin binding buffer. Then, 5 μL AnnexinV-FITC and 1 μL PI dye were added into the cell suspension and incubated for 30 min, avoiding light. Apoptosis was monitored by flow cytometry.

#### Determination of free radical formation

Cells were grown on a glass coverslip, and then treated with Ni NPs alone or Ni NPs + EGCG for 24 h. Then, the cells were immobilized with 90% ethanol on ice. A 200 μL solution containing H2DCFDA (5 μM), DHE (2 μM), and Hoechst33258 (3 μM) were added onto the cover slip. After 1 h incubation in the dark on ice, cells were washed gently 3 times with 4°C sterile PBS. Finally, a drop of Flu-G was dropped onto each glass coverslip, covered with a glass slide, and sealed around the edges. The images of intracellular ROS generation were captured with a confocal laser scanning microscope.

The intracellular ROS levels were detected by a reactive oxygen species assay kit. The JB6 cells were maintained at a density of 10,000 cells/well in a 96-well black plate. After different treatments, cells were washed 3 times with 37°C sterile PBS, and then incubated with 10 μM H2DCFDA on an incubator shaker at 37°C for 25 min. The fluorescence distribution was detected by a fluorospectrophotometer at an excitation wavelength of 488 nm and an emission wavelength of 525 nm.

#### Detection of luciferase activity

The JB6 cells transfected with AP-1 or NF-κB gene reporters (a gift from NIOSH) were used to detect luciferase activity of AP-1 or NF-κB. A 2 mL cell suspension (1×10^5^ cell/mL) was seeded into a 24-well plate and maintained at standard culture conditions for 24 h. Cells were incubated in 0.1% FBS DMEM at standard culture conditions (37 ℃, 80% humidified air and 5% CO_2_) for 24 h. Then, cells were treated with Ni NPs alone or Ni NPs + EGCG for 24 h. Cells exposed to 20 nM TPA were used as a positive control. Cells were lysed with 1×cell lysis buffer (120 μL) for 1 h and then the lysate was centrifuged for 20 min at 12,000 rpm, 4°C. A sample of supernatant (20 μL) and Promega test reagents (100 μL: luciferase assay substrate mixed with luciferase assay buffer) were transferred into dedicated centrifuge tubes. After mixing well, the luciferase activity of AP-1 or NF-κB was detected following the manufacturer’s instruction.

#### Western blot analysis

After seeding into two 6-well plates and cultured for 24 h, cells were treated Ni NPs alone or Ni NPs + EGCG for 24 h. Then, cells were washed twice with cold PBS. A 60 μL mixture of EDTA-free, PMSF and NP-40 was added into each well to lyse cells on ice for 1 h, and then the lysate was centrifuged at 12000 rpm, 4°C for 25 min. Protein concentrations in the supernatants were determined using the bicinchoninic acid method. Equal amounts of protein were separated by 6% and 10% polyacrylamide gels. Immunoblots for expressions of AP-1, NF-κB, JNK, p-JNK, ERK1/2, p-ERK1/2, p38, p-p38, and GAPDH were detected. Equal amounts of protein were ensured by measuring GAPDH. A gel imaging processing system was used for western blot analysis.

#### Statistical analysis

Every experiment was performed three or more times and the data were presented as means ± standard errors ($\bar{x}$ ± SE) of the number of experiments/samples. Data were analyzed using *T*-test or One-way ANOVA analysis by SPSS16.0 and SAS9.1. Significance was set at *P*≤0.05.

## Results

### Physical characteristics of the Ni NPs

**Fig 1:**
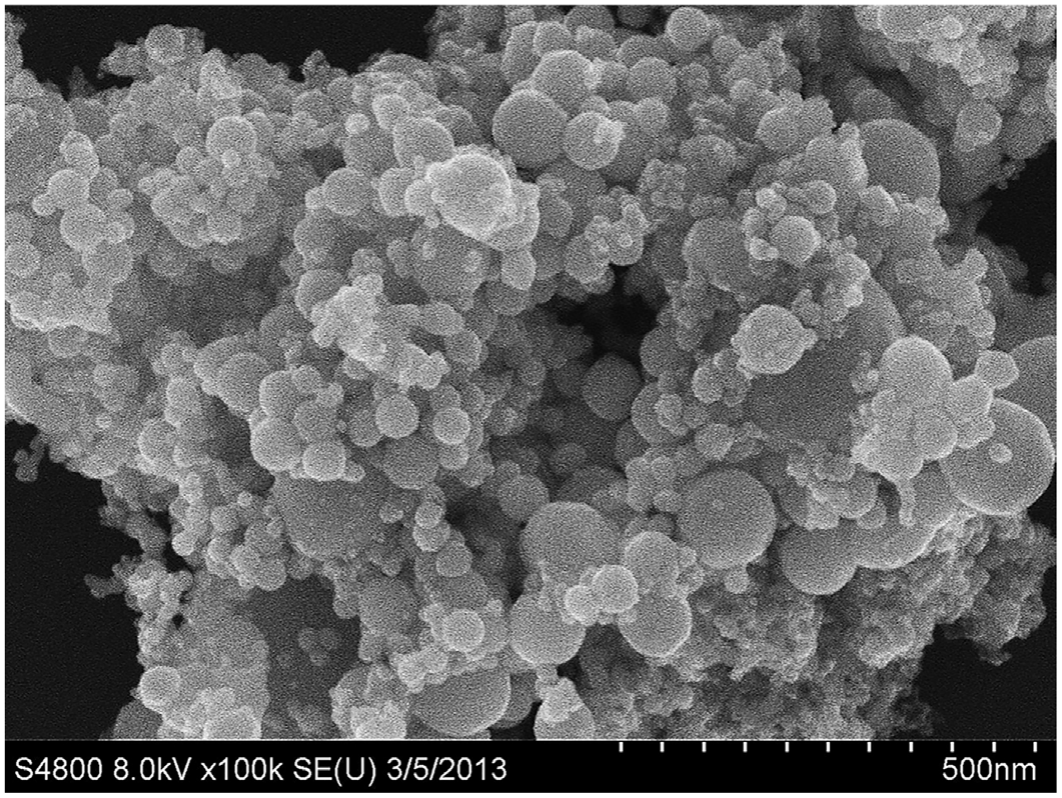
Image of nickel nanoparticles captured by scanning electron microscopy.

The results detected by SEM showed that the size of Ni NPs was 40.50 ± 18.6 nm, and the mean surface area was 28 m^2^/g (Table 1, Fig 1).

**Table 1:** Physical characteristic of Ni NPs

Sample	Particle size (nm,$\bar{x}$ ± SE)	Surface area (m2/g)
Ni NPs^a^	40.50 ± 18.6	28

^a^Ni NPs, nickel nanoparticles

### Cell viability and morphological changes

**Fig 2:**
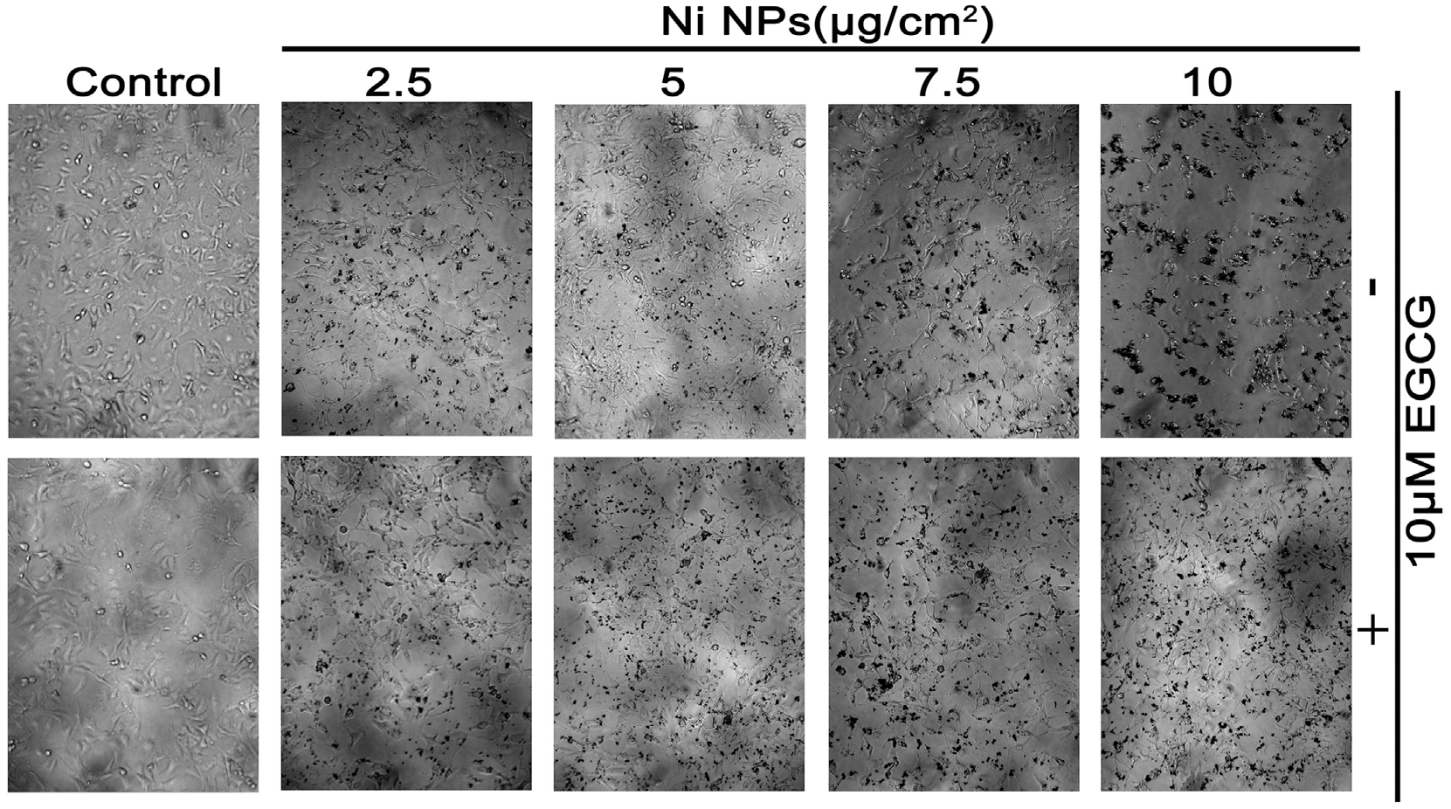
Morphological changes after cells were treated with Ni NPs alone or Ni NPs + EGCG. Note: Magnification of the light microscope was at 20×. Abbreviations: Ni NPs, nickel nanoparticles; EGCG, epigallocatechin-3-gallate.

**Fig 3:**
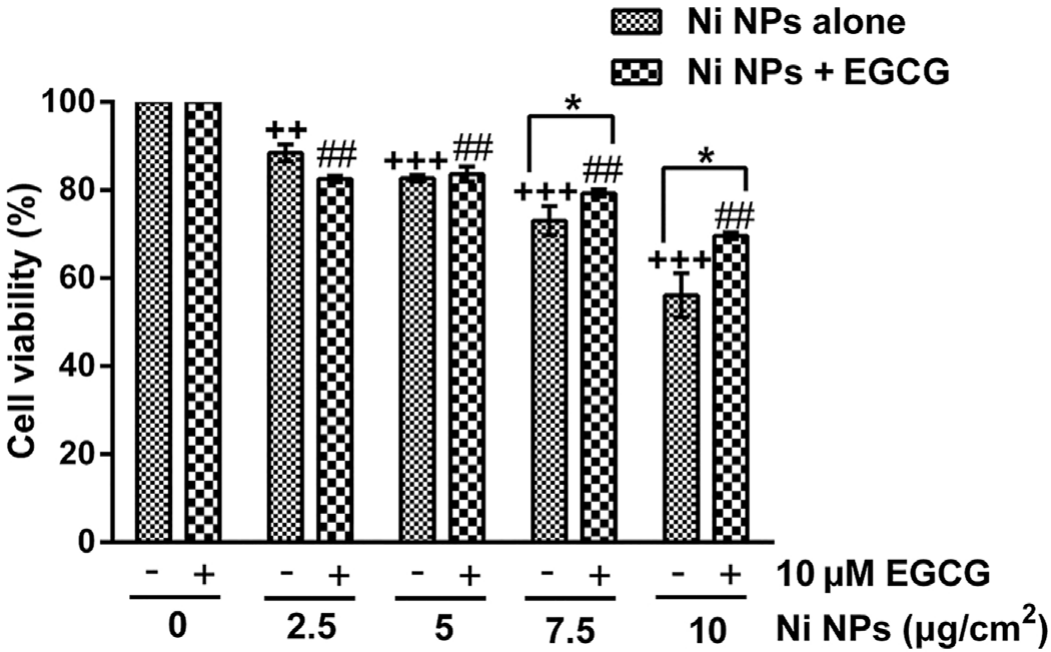
Cell viability after cells were treated with Ni NPs alone or Ni NPs + EGCG. Note: *P<0.05, Ni NPs alone compared with Ni NPs + EGCG; ++ *P*<0.01, +++ *P*<0.001, compared with control—(without any treatment); ## *P*<0.01, ### *P*<0.001, compared with control + 10 μM EGCG; error bars, SE. Abbreviations: Ni NPs, nickel nanoparticles; EGCG, epigallocatechin-3-gallate.

Significant cell viability reduction and toxic morphological changes were observed in Figs 2 and 3. Following 7.5 and 10 μg/cm^2^ Ni NPs exposure, the number of surviving cells showed a significant difference between Ni NPs alone and Ni NPs + EGCG treatments.

### Cell cycle analysis

**Fig 4:**
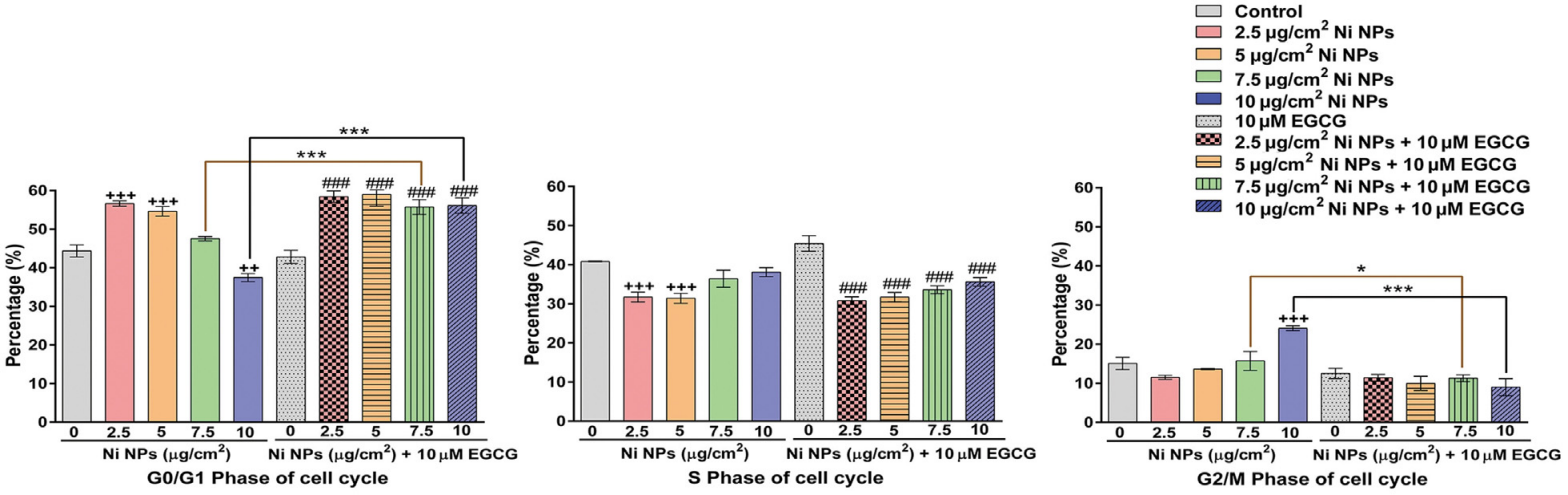
Cell cycle analysis after cells were treated with Ni NPs alone or Ni NPs + EGCG. Notes: * *P*<0.05, *** *P*<0.001, Ni NPs alone compared with Ni NPs + EGCG; ++ *P*<0.01, +++ *P*<0.001, compared with control—(without any treatment); ###, *P*<0.001, compared with control + 10 μM EGCG; error bars, SE. Abbreviations: Ni NPs, nickel nanoparticles; EGCG, epigallocatechin-3-gallate.

After 2.5 and 5 μg/cm^2^ Ni NPs treatment alone, obvious G0/G1 phase arrest was detected. With each increase of Ni NPs concentration, G0/G1 phase arrest declined accompanying with a significant increase of G2/M phase arrest. Addition of 10 μM EGCG resulted in a significantly increase in G0/G1 phase arrest and a decrease in G2/M phase arrest in the 7.5 and 10 μg/cm^2^ Ni NPs treatment groups. (As shown in Fig 4 and S1 Fig.)

### Cell apoptosis

**Fig 5:**
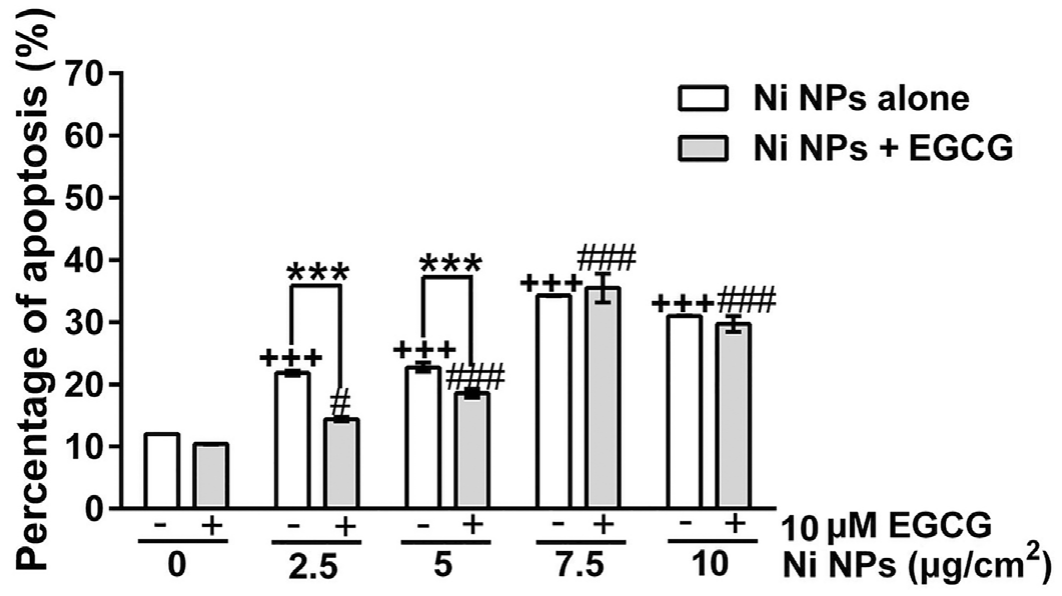
Comparison of apoptotic cell number^§^ after cells were treated with Ni NPs alone or Ni NPs + EGCG. Notes: ^§^including early and late-stage apoptotic cells; *** *P*<0.001, Ni NPs alone compared with Ni NPs + EGCG; +++ *P*<0.001, compared with control—(without any treatment); # *P*<0.05, ### *P*<0.001, compared with control + 10 μM EGCG; error bars, SE. Abbreviations: Ni NPs, nickel nanoparticles; EGCG, epigallocatechin-3-gallate.

As shown in Fig 5 and S2 Fig, with increase Ni NPs concentration, apoptotic cells increased and 10 μM EGCG could only significantly inhibit cell apoptosis in the 2.5 and 5 μg/cm^2^ Ni NPs treatment groups.

### ROS generation

**Fig 6:**
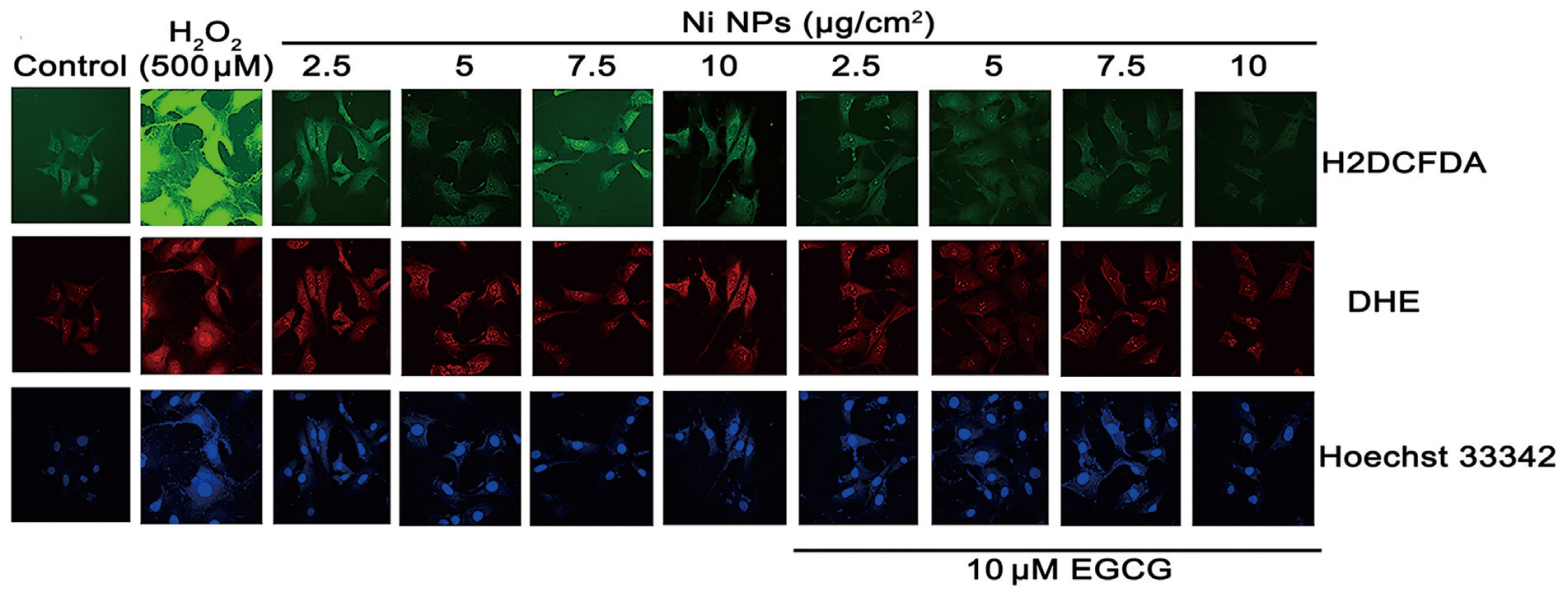
Oxidative stress staining after JB6 cells were treated with Ni NPs alone or Ni NPs + EGCG. Notes: H2DCFDA (green) and DHE (red) are used for staining general ROS and •O_2_^-^ produced in the intact cells, respectively. Hoechst 33258 (blue) is a nucleic acid stain. 500 μM H_2_O_2_ was used as a positive control for ROS generation. Abbreviations: Ni NPs, nickel nanoparticles; EGCG, epigallocatechin-3-gallate; H2DCFDA, 2',7'-dichlorodihydrofluorescein diacetate; DHE, dihydroethidium; ROS, reactive oxygen species.

**Fig 7:**
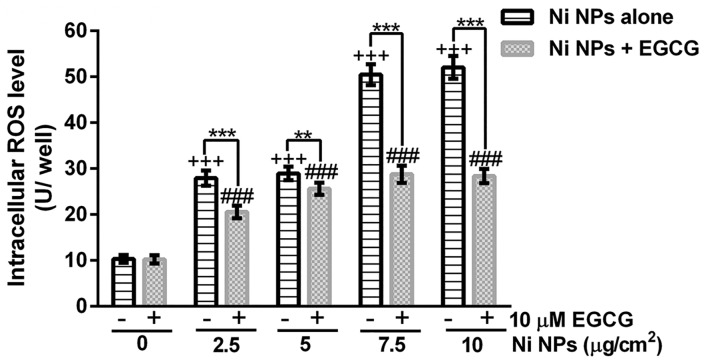
ROS levels after JB6 cells were treated with EGCG alone, Ni NPs alone or Ni NPs + EGCG. Notes: ** *P*<0.01, *** *P*<0.001, Ni NPs alone compared with Ni NPs + EGCG; +++ *P*<0.001, compared with control (without any treatment); ###, *P*<0.001, compared with control + 10 μM EGCG; error bars, SE. Abbreviations: Ni NPs, nickel nanoparticles; EGCG, epigallocatechin-3-gallate.

The results showed that Ni NPs induced intracellular ROS generation in a dose-dependent manner and the intracellular ROS could be significantly reduced by EGCG (Figs 6 and 7).

### Luciferase activities of AP-1 and NF-κB

**Fig 8:**
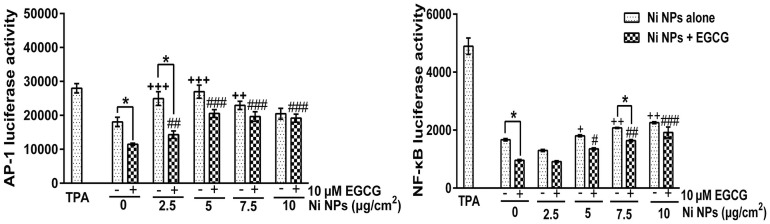
Luciferase activity of AP-1 and NF-κB after JB6 cells were treated with Ni NPs alone or Ni NPs + EGCG. Notes: *P<0.05, Ni NPs alone compared with Ni NPs + EGCG; + *P*<0.05, ++ *P*<0.01, +++ *P*<0.001, compared with control—(without any treatment); # *P*<0.01, ## *P*<0.01, ### *P*<0.001, compared with control + 10 μM EGCG. 20 nM TPA was set as a positive control. Abbreviations: Ni NPs, nickel nanoparticles; EGCG, epigallocatechin-3-gallate; TPA, phorbol ester.

As shown in Fig 8, Ni NPs alone could induce AP-1 and NF-κB luciferase activity. A supplement of 10 μM EGCG showed a significant inhibition on Ni NPs-induced AP-1 and NF-κB luciferase activity, especially in the 2.5 and 5 μg/cm^2^ Ni NPs treatment groups.

### MAPK signaling protein expressions

**Fig 9:**
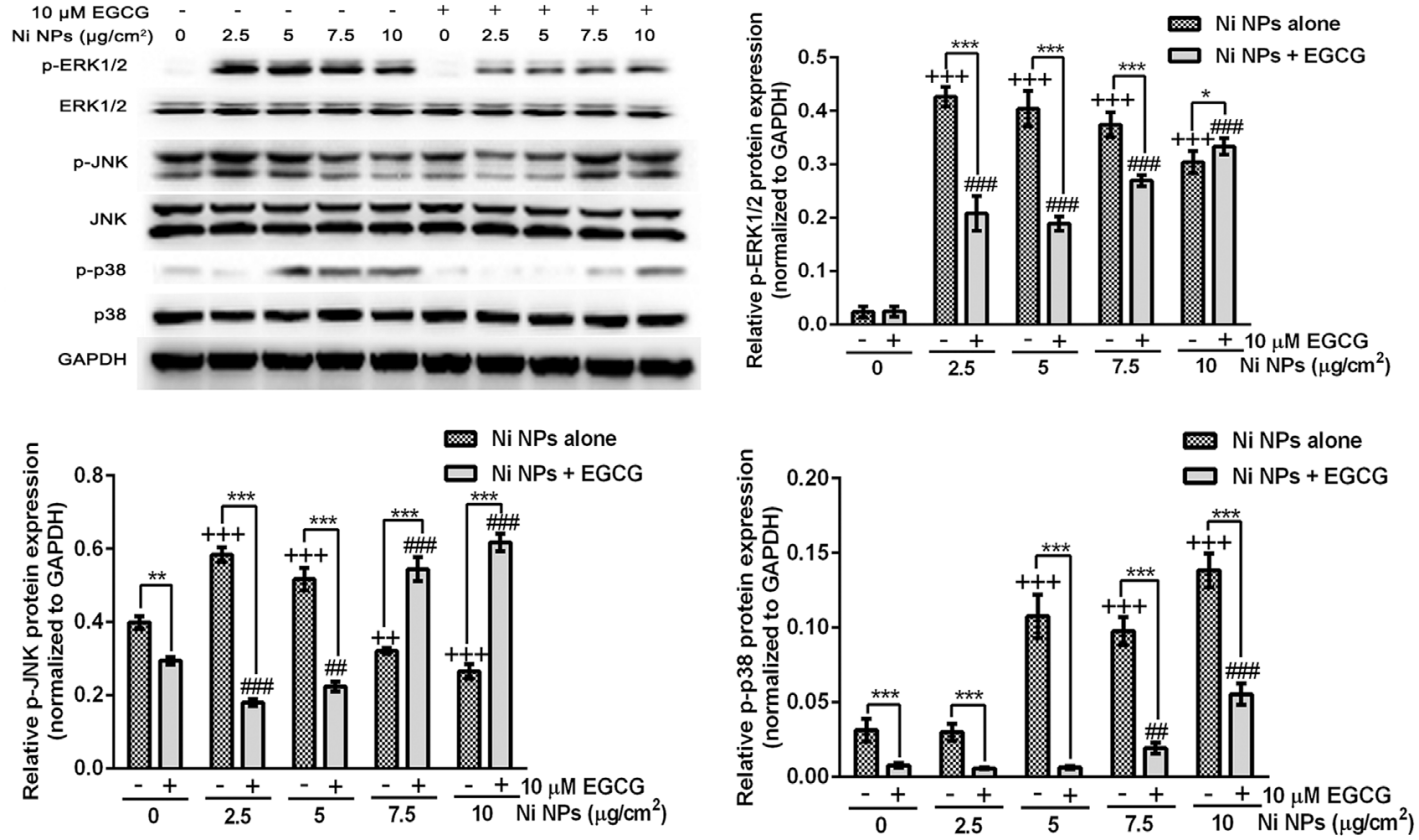
Expression levels of MAPK signal pathway proteins after cells were treated with Ni NPs alone or Ni NPs + EGCG. Notes: *P<0.05, **P<0.01, ***P<0.001, Ni NPs alone compared with Ni NPs + EGCG; ++ *P*<0.01, +++ *P*<0.001, compared with control—(without any treatments); ## *P*<0.01, ### *P*<0.001, compared with control + 10 μM EGCG. Abbreviations: Ni NPs, nickel nanoparticles; EGCG, epigallocatechin-3-gallate.

As shown in Fig 9, the inhibitory effects of EGCG on Ni NPs-induced p-ERK1/2, p-JNK and p-p38 protein up-regulation were only observed in the 2.5 and 5 μg/cm^2^ Ni NPs treatment groups.

## Discussion

Our results indicate that Ni NPs caused a dose-dependent decrease in cell viability accompanying with a significant increase in intracellular ROS generation and apoptosis. A supplement of 10 μM EGCG shows a definite inhibition on Ni NPs-induced toxicity, especially in the 2.5 and 5 μg/cm^2^ groups. Also, 10 μM EGCG showed a significant inhibition on AP-1 and NF-κB luciferase activity, as well as on MAPK signaling protein expressions (p-ERK1, p-JNK or p-p38) in the 2.5 and 5 μg/cm^2^ groups.

The cell cycle can be divided into four stages, known as G0/G1 (the early stage of DNA synthesis), G2 (the later stage of DNA synthesis), M (the stage of mitosis), and S (the stage of DNA synthesis). To our knowledge, the G0/G1 phase is the key that starts the cell cycle. If the cell cycle is arrested at G0/G1 phase, cells will not be able to enter into the stage of mitosis and cell proliferation, eventually leading to apoptosis. The G2/M phase arrest can be caused by physical and chemical factors inducing DNA damage [22]. Ahmad *et al* reported that Ni NPs (28 nm; concentration range, 25–100 μg/mL) induced oxidative stress in a dose-dependent manner accompanying with ROS generation, subG1 arrest and DNA damage [23]. Similar to the results above, we found that 2.5 and 5 μg/cm^2^ Ni NPs could induce the G0/G1 phase arrest, and 7.5 and 10 μg/cm^2^ Ni NPs could induce the G2/M phase arrest. These results suggest that 2.5 and 5 μg/cm^2^ Ni NPs induced cell apoptosis, whereas 7.5 and 10 μg/cm^2^ Ni NPs might cause cell necrosis through DNA damage. Addition of 10 μM EGCG can result in G0/G1 phase arrest in the 7.5 and 10 μg/cm^2^ groups. This may suggest that EGCG can reduce oxidative stress-mediated DNA damage and cell necrosis.

Apoptosis is an initiative action of cells to implement programmed death [24]. It is caused by a series of physiological and pathological signals. Under the regulation of the death related genes, death receptor pathways are activated, including the membrane receptor pathway, cytochrome c pathway and caspase pathway [25–27]. Our results showed that Ni NPs induced cell apoptosis in a dose-dependent manner at low concentrations (2.5 and 5 μg/cm^2^). The inhibitory effect of EGCG on Ni NPs-induced cell apoptosis was only observed in the 2.5 and 5 μg/cm^2^ groups. This implies that 10 μM EGCG could only quench the apoptotic effects at low concentration of Ni NPs.

Normally, intracellular ROS generation and quenching are in a dynamic balance state. Harmful factors may break this balance, resulting in excessive generation of ROS beyond the scavenging ability of intracellular antioxidant system, and then inducing DNA damage and abnormal expression of proteins. As an antioxidant, the effect of EGCG on ROS generation is biphasic. The low dose of EGCG can reduce the level of intracellular ROS. However, the high dose of EGCG can induce ROS generation [28]. This study showed that Ni NPs could induce intracellular ROS generation in a dose-dependent manner and EGCG could significantly reduce it. Meanwhile, 10 μM EGCG showed no significant ROS generation to JB6 cells. These results suggested that oxidative stress injury played an important role in Ni NPs-induced cell apoptosis and EGCG could inhibit the toxicity by removing excessive ROS.

AP-1 is a common intracellular transcription activator [29]. Previous studies have shown that AP-1 participates in many important cellular activities, including cell differentiation, cell proliferation and apoptosis [30,31]. In addition, the up-regulation of AP-1 has also been found to be related to tumorigenesis [32,33]. Similar to AP-1, NF-κB has also been found to be related to tumorigenesis, inflammation and autoimmune diseases [34–36]. In previous studies, we found that compared to fine nickel particles, Ni NPs are more likely to up-regulate the expression levels of AP-1 and NF-κB. In this study, we found that a supplement of 10 μM EGCG could partially down-regulate the expression levels of AP-1 and NF-κB. These results suggest that EGCG might have inhibitory effects on Ni NPs-induced carcinogenicity.

To explore the mechanism of the changes of AP-1 and NF-κB, we further detected the expression levels of MAPK signal proteins. The MAPK signaling pathways family includes ERKl/2, JNK, p38, and ERK5, etc. They can be activated by UV, growth factors, cytokines, and DNA damaging agents [37,38]. The MAPK signaling pathways are involved in cell differentiation, apoptosis and inflammation. ERK1/2 can be activated by phosphorylation to regulate some nuclear transcription factors such as c-fos, c-Jun, Elk-1, c-myc, and ATF2, which are involved in cell proliferation and cell differentiation. JNK can also be activated by phosphorylation to activate c-Jun and then to up-regulate the transcription activity of AP-1. After p38 has been activated by phosphorylation, IκB can be phosphorylated and cleaved, thus leading to depolymerization of IκB and NF-κB, and eventually resulting in nuclear migration and the releasing and activation of NF-κB. In this study, Ni NPs induced significant up-regulation of protein expressions of p-JNK, p-ERK1/2 and p-p38. EGCG could inhibit the up-regulation of protein expressions of p-JNK, p-ERK1/2 and p-p38, especially in the 2.5 and 5 μg/cm^2^ groups. This was consistent with the fact that EGCG could inhibit the up-regulation of AP-1 and NF-κB luciferase activity induced by Ni NPs.

Taken together, our results suggest that Ni NPs could induce intracellular ROS generation and result in up-regulation of the transcriptional level of AP-1 and NF-κB through the MAPK signaling pathways which might be contributed to cell apoptosis, necrosis and carcinogenesis. Inhibition of EGCG on Ni NPs-induced cytotoxicity in JB6 cells might be through the MAPK signaling pathways indicating that EGCG might be useful in preventing Ni NPs-induced toxicity.

## Limitation

Although EGCG is an antioxidant, it could produce ROS when added to the cell culture medium for the reason of auto-oxidation. In this study, we found that 10 μM EGCG could reduce Ni NPs-induced toxicity and no obvious ROS generation under its treatment alone was observed. Previous evidence showed that addition of SOD and catalase to the medium simultaneously could prevent the generation of ROS induced by the auto-oxidation of EGCG [39]. Therefore, future studies will be necessary to determine whether these enzymes can enhance the inhibitory effects of EGCG on Ni NPs-induced toxicity. Besides, EGCG inhibition on Ni NPs-induced toxicity was only evaluated by *in vitro* experiments in this study. The data obtained from our *in vitro* studies may not be enough to thoroughly evaluate the inhibitory effect of EGCG on Ni NPs-induced toxicities. The study of Zhou H *et al* indicates that the preventive mechanism of EGCG *in vivo* is obviously different from that found *in vitro* [40]. Thus, further *in vivo* experiments will be absolutely necessary to explore the toxicokinetics effect of Ni NPs and the pharmacodynamics effect of EGCG.

## Supporting Information

S1 Fig Cell cycle analysis after cells were treated with Ni NPs alone or Ni NPs + EGCG. Abbreviations: Ni NPs, nickel nanoparticles; EGCG, epigallocatechin-3-gallate
Note: S1 Fig is the result of cell cycle analysis detected by flow cytometry to support Fig 4.
(TIF)

S2 Fig Cell apoptotic induction after treatments with Ni NPs alone or Ni NPs + EGCG. Abbreviations: Ni NPs, nickel nanoparticles; EGCG, epigallocatechin-3-gallate
Notes: S2 Fig is the result of cell apoptotic detected by flow cytometry to support Fig 5. The upper left quadrant (UL) was PI+/Annexin V-, representing mechanical-induced cell damage and late cell death; the lower left quadrant (LL) was PI-/Annexin V-, representing normal cells; the upper right quadrant (UR) was PI+/Annexin V+, representing late-stage apoptotic cells; the lower right quadrant (LR) was PI-/Annexin V+, representing the early-stage apoptotic cells.
(TIF)

## References

[1] Scuri M, Chen BT, Castranova V, Reynolds JS, Johnson VJ, Samsell L, et al Effects of titanium dioxide nanoparticle exposure on neuroimmune responses in rat airways. J Toxicol Environ Health A. 2010;73(20):1353–69. 10.1080/15287394.2010.497436 20818535 PMC3655524

[2] Zhang Q, Kusaka Y, Sato K, Nakakuki K, Kohyama N, Donaldson K. Differences in the extent of inflammation caused by intratracheal exposure to three ultrafine metals: role of free radicals. J Toxicol Environ Health A. 1998;53(6):423–38. 9537280 10.1080/009841098159169

[3] Zhao J, Shi X, Castranova V, Ding M. Occupational toxicology of nickel and nickel compounds. J Environ Pathol Toxicol Oncol. 2009;28(3):177–208. 19888907 10.1615/jenvironpatholtoxicoloncol.v28.i3.10

[4] Nielsen NH, Menne T, Kristiansen J, Christensen JM, Borg L, Poulsen LK. Effects of repeated skin exposure to low nickel concentrations: a model for allergic contact dermatitis to nickel on the hands. Br J Dermatol. 1999;141(4):676–82. 10583115 10.1046/j.1365-2133.1999.03106.x

[5] Kasprzak KS, Sunderman FW Jr., Salnikow K. Nickel carcinogenesis. Mutat Res. 2003;533(1–2):67–97. 14643413 10.1016/j.mrfmmm.2003.08.021

[6] Permenter MG, Lewis JA, Jackson DA. Exposure to nickel, chromium, or cadmium causes distinct changes in the gene expression patterns of a rat liver derived cell line. PloS one. 2011;6(11):e27730 10.1371/journal.pone.0027730 22110744 PMC3218028

[7] Magaye R, Zhou Q, Bowman L, Zou B, Mao G, Xu J, et al Metallic nickel nanoparticles may exhibit higher carcinogenic potential than fine particles in JB6 cells. PloS one. 2014;9(4):e92418 10.1371/journal.pone.0092418 24691273 PMC3972196

[8] Park S, Lee YK, Jung M, Kim KH, Chung N, Ahn EK, et al Cellular toxicity of various inhalable metal nanoparticles on human alveolar epithelial cells. Inhal Toxicol. 2007;19 Suppl 1:59–65. 17886052 10.1080/08958370701493282

[9] Cameron KS, Buchner V, Tchounwou PB. Exploring the molecular mechanisms of nickel-induced genotoxicity and carcinogenicity: a literature review. Rev Environ Health. 2011;26(2):81–92. 21905451 10.1515/reveh.2011.012 PMC3172618

[10] Zhao J, Bowman L, Zhang X, Shi X, Jiang B, Castranova V, et al Metallic nickel nano- and fine particles induce JB6 cell apoptosis through a caspase-8/AIF mediated cytochrome c-independent pathway. Journal of Nanobiotechnology. 2009;7:2 10.1186/1477-3155-7-2 19379505 PMC2673202

[11] Pietruska JR, Liu X, Smith A, McNeil K, Weston P, Zhitkovich A, et al Bioavailability, intracellular mobilization of nickel, and HIF-1alpha activation in human lung epithelial cells exposed to metallic nickel and nickel oxide nanoparticles. Toxicol Sci. 2011;124(1):138–48. 10.1093/toxsci/kfr206 21828359 PMC3196652

[12] Hansen T, Clermont G, Alves A, Eloy R, Brochhausen C, Boutrand JP, et al Biological tolerance of different materials in bulk and nanoparticulate form in a rat model: sarcoma development by nanoparticles. J R Soc Interface. 2006;3(11):767–75. 17015296 10.1098/rsif.2006.0145 PMC1885365

[13] Phillips JI, Green FY, Davies JC, Murray J. Pulmonary and systemic toxicity following exposure to nickel nanoparticles. Am J Ind Med. 2010;53(8):763–7. 10.1002/ajim.20855 20623660

[14] Zhang X, He F, Yang J, Chen ZS. Protective effects of epigallocatechin-3-gallate on intestinal is chemia reperfusion injury through enhanced activation of PI3K/Akt pathway in rats. J Huazhong Univ Sci Technolog Med Sci. 2015;35(3):378–83. 10.1007/s11596-015-1441-2 26072077

[15] Kim JY, Choi JY, Lee HJ, Byun CJ, Park JH, Park JH, et al The Green Tea Component (-)-Epigallocatechin-3-Gallate Sensitizes Primary Endothelial Cells to Arsenite-Induced Apoptosis by Decreasing c-Jun N-Terminal Kinase-Mediated Catalase Activity. PloS one. 2015;10(9):e0138590 10.1371/journal.pone.0138590 26375285 PMC4574201

[16] Fang MZ, Wang Y, Ai N, Hou Z, Sun Y, Lu H, et al Tea polyphenol (-)-epigallocatechin-3-gallate inhibits DNA methyltransferase and reactivates methylation-silenced genes in cancer cell lines. Cancer Res. 2003;63(22):7563–70. 14633667

[17] Ye T, Zhen J, Du Y, Zhou JK, Peng A, Vaziri ND, et al Green tea polyphenol (-)-epigallocatechin-3-gallate restores Nrf2 activity and ameliorates crescentic glomerulonephritis. PloS one. 2015;10(3):e0119543 10.1371/journal.pone.0119543 25785827 PMC4364748

[18] Liao S, Umekita Y, Guo J, Kokontis JM, Hiipakka RA. Growth inhibition and regression of human prostate and breast tumors in athymic mice by tea epigallocatechin gallate. Cancer Lett. 1995;96(2):239–43. 7585463 10.1016/0304-3835(95)03948-v

[19] Tsang WP, Kwok TT. Epigallocatechin gallate up-regulation of miR-16 and induction of apoptosis in human cancer cells. J Nutr Biochem. 2010;21(2):140–6. 10.1016/j.jnutbio.2008.12.003 19269153

[20] Kuo PL, Lin CC. Green tea constituent (-)-epigallocatechin-3-gallate inhibits Hep G2 cell proliferation and induces apoptosis through p53-dependent and Fas-mediated pathways. J Biomed Sci. 2003;10(2):219–27. 12595758 10.1007/BF02256057

[21] Singh BN, Shankar S, Srivastava RK. Green tea catechin, epigallocatechin-3-gallate (EGCG): mechanisms, perspectives and clinical applications. Biochem Pharmacol. 2011;82(12):1807–21. 10.1016/j.bcp.2011.07.093 21827739 PMC4082721

[22] Taylor WR, Stark GR. Regulation of the G2/M transition by p53. Oncogene. 2001;20(15):1803–15. 11313928 10.1038/sj.onc.1204252

[23] Ahmad J, Alhadlaq HA, Siddiqui MA, Saquib Q, Al-Khedhairy AA, Musarrat J, et al Concentration-dependent induction of reactive oxygen species, cell cycle arrest and apoptosis in human liver cells after nickel nanoparticles exposure. Environ Toxicol. 2015;30(2):137–48. 10.1002/tox.21879 23776134

[24] Kerr JF, Wyllie AH, Currie AR. Apoptosis: a basic biological phenomenon with wide-ranging implications in tissue kinetics. Br J Cancer. 1972;26(4):239–57. 4561027 10.1038/bjc.1972.33 PMC2008650

[25] Egger L, Madden DT, Rheme C, Rao RV, Bredesen DE. Endoplasmic reticulum stress-induced cell death mediated by the proteasome. Cell Death Differ. 2007;14(6):1172–80. 17396132 10.1038/sj.cdd.4402125 PMC2748804

[26] Qiu Q, Wang Z, Jiang JM, Li DD. Effect of lidamycin on mitochondria initiated apoptotic pathway in human cancer cells. Yao Xue Xue Bao. 2007;42(2):132–8. 17518039

[27] Eskes R, Desagher S, Antonsson B, Martinou JC. Bid induces the oligomerization and insertion of Bax into the outer mitochondrial membrane. Mol Cell Biol. 2000;20(3):929–35. 10629050 10.1128/mcb.20.3.929-935.2000 PMC85210

[28] Kucera O, Mezera V, Moravcova A, Endlicher R, Lotkova H, Drahota Z, et al In vitro toxicity of epigallocatechin gallate in rat liver mitochondria and hepatocytes. Oxidative Medicine and Cellular Longevity. 2015;2015:476180 10.1155/2015/476180 25918582 PMC4397056

[29] Zhang Y, Liu L. Progress in activator protein-1 and the allergic diseases. Journal of Medical Postgraduates. 2007;20(2):199–202.

[30] Karin M, Liu Z, Zandi E. AP-1 function and regulation. Curr Opin Cell Biol. 1997;9(2):240–6. 9069263 10.1016/s0955-0674(97)80068-3

[31] Shaulian E, Karin M. AP-1 as a regulator of cell life and death. Nat Cell Biol. 2002;4(5):E131–6. 11988758 10.1038/ncb0502-e131

[32] Jiang XH, Wong BC, Lin MC, Zhu GH, Kung HF, Jiang SH, et al Functional p53 is required for triptolide-induced apoptosis and AP-1 and nuclear factor-kappaB activation in gastric cancer cells. Oncogene. 2001;20(55):8009–18. 11753684 10.1038/sj.onc.1204981

[33] Yao KS, Xanthoudakis S, Curran T, O'Dwyer PJ. Activation of AP-1 and of a nuclear redox factor, Ref-1, in the response of HT29 colon cancer cells to hypoxia. Mol Cell Biol. 1994;14(9):5997–6003. 8065332 10.1128/mcb.14.9.5997-6003.1994 PMC359125

[34] Elwood PC, Gallagher AM, Duthie GG, Mur LA, Morgan G. Aspirin, salicylates, and cancer. Lancet. 2009;373(9671):1301–9. 10.1016/S0140-6736(09)60243-9 19328542

[35] Baldwin AS. Control of oncogenesis and cancer therapy resistance by the transcription factor NF-kappaB. J Clin Invest. 2001;107(3):241–6. 11160144 10.1172/JCI11991 PMC199203

[36] Pikarsky E, Porat RM, Stein I, Abramovitch R, Amit S, Kasem S, et al NF-kappaB functions as a tumour promoter in inflammation-associated cancer. Nature. 2004;431(7007):461–6. 15329734 10.1038/nature02924

[37] Junttila MR, Li SP, Westermarck J. Phosphatase-mediated crosstalk between MAPK signaling pathways in the regulation of cell survival. Faseb J. 2008;22(4):954–65. 18039929 10.1096/fj.06-7859rev

[38] Wang Z, Yang H, Tachado SD, Capo-Aponte JE, Bildin VN, Koziel H, et al Phosphatase-mediated crosstalk control of ERK and p38 MAPK signaling in corneal epithelial cells. Invest Ophthalmol Vis Sci. 2006;47(12):5267–75. 17122112 10.1167/iovs.06-0642

[39] Wang H, Bian S, Yang CS. Green tea polyphenol EGCG suppresses lung cancer cell growth through upregulating miR-210 expression caused by stabilizing HIF-1alpha. Carcinogenesis. 2011;32(12):1881–9. 10.1093/carcin/bgr218 21965273 PMC3220612

[40] Zhou H, Chen JX, Yang CS, Yang MQ, Deng Y, Wang H. Gene regulation mediated by microRNAs in response to green tea polyphenol EGCG in mouse lung cancer. BMC genomics. 2014;15 Suppl 11:S3 10.1186/1471-2164-15-S11-S3 25559244 PMC4304179

