# Correction: Inhibition of Nickel Nanoparticles-Induced Toxicity by Epigallocatechin-3-Gallate in JB6 Cells May Be through Down-Regulation of the MAPK Signaling Pathways

**DOI:** 10.1371/journal.pone.0154978

**Published:** 2016-04-28

**Authors:** Yuanliang Gu, Yafei Wang, Qi Zhou, Linda Bowman, Guochuan Mao, Baobo Zou, Jin Xu, Yu Liu, Kui Liu, Jinshun Zhao, Min Ding

There is an error in the Funding section. The complete, correct Funding Statement is as follows:

This work was partly supported by the National Nature Science Foundation of China (Grant No. 81273111), the Ningbo Scientific Project (Grant No. 2012C5019), the Science Technology Department of Zhejiang Province (2015C33148 and 2015C37117) and the Scientific Innovation Team Project of Ningbo (No. 2011B82014). The authors also gratefully acknowledge the support of the K.C. Wong Magna Fund in Ningbo University. The funders had no role in study design, data collection and analysis, decision to publish, or preparation of the manuscript.
