# Supplementary figures and images for: Inhibition of Nickel Nanoparticles-Induced Toxicity by Epigallocatechin-3-Gallate in JB6 Cells May Be through Down-Regulation of the MAPK Signaling Pathways
Source: PLoS One. 2016 Mar 4;11(3):e0150954. doi: 10.1371/journal.pone.0150954 (PMC4778769; doi:10.1371/journal.pone.0150954)

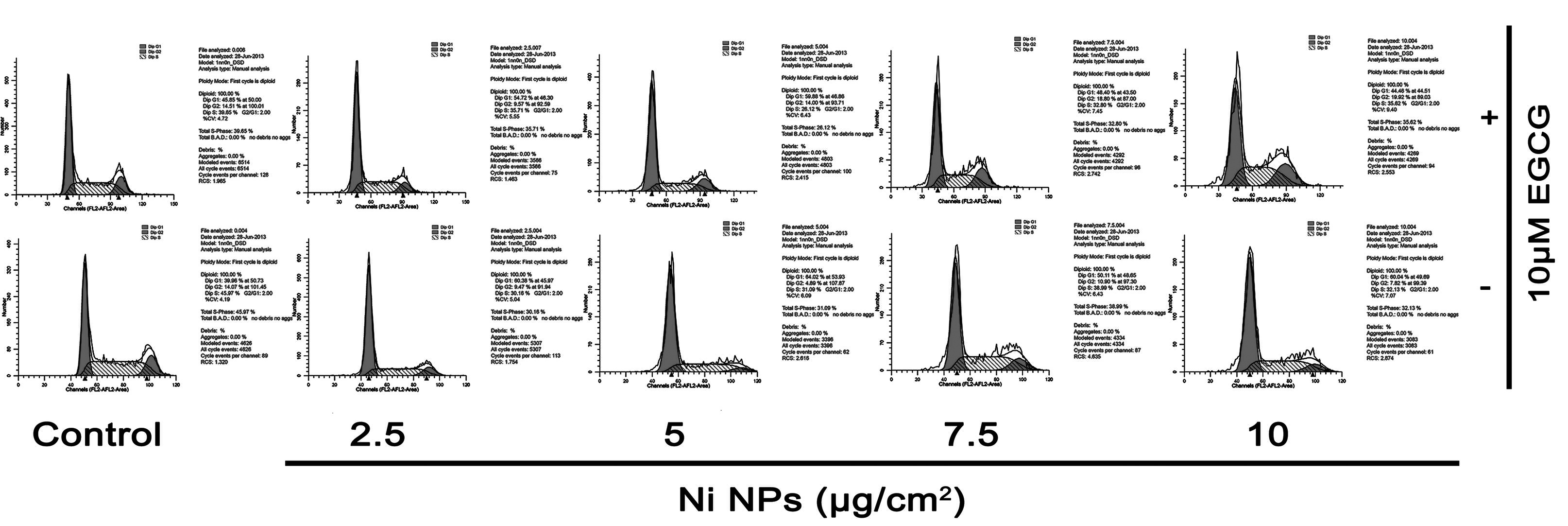

Supplement: S1 Fig — Abbreviations: Ni NPs, nickel nanoparticles; EGCG, epigallocatechin-3-gallate Note: S1 Fig is the result of cell cycle analysis detected by flow cytometry to support Fig 4. (TIF) [file pone.0150954.s001.tif]

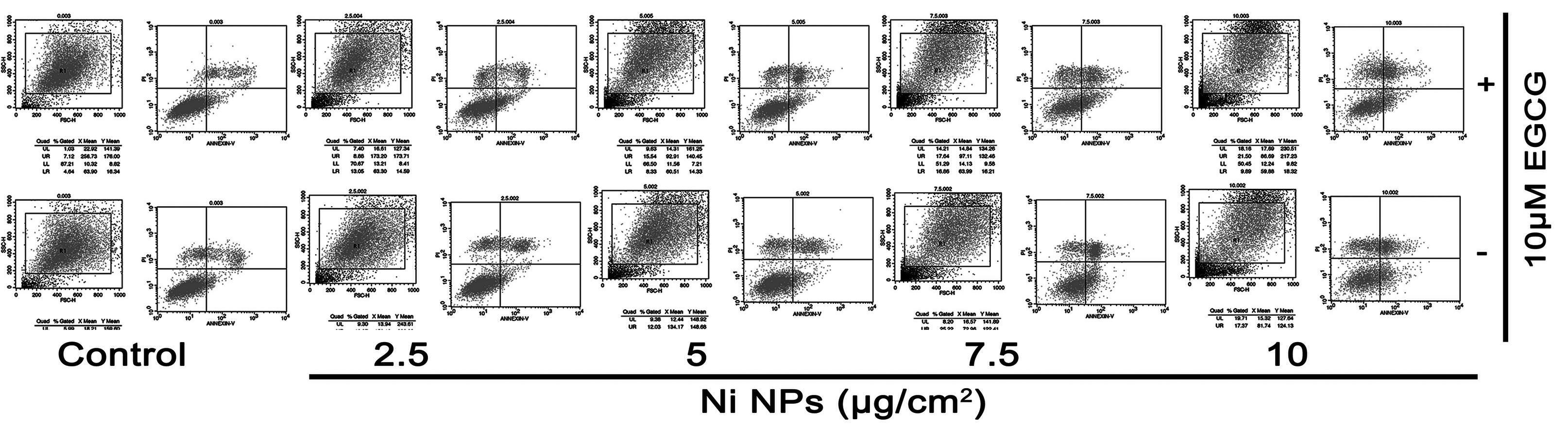

Supplement: S2 Fig — Abbreviations: Ni NPs, nickel nanoparticles; EGCG, epigallocatechin-3-gallate Notes: S2 Fig is the result of cell apoptotic detected by flow cytometry to support Fig 5. The upper left quadrant (UL) was PI+/Annexin V-, representing mechanical-induced cell damage and late cell death; the lower left quadrant (LL) was PI-/Annexin V-, representing normal cells; the upper right quadrant (UR) was PI+/Annexin V+, representing late-stage apoptotic cells; the lower right quadrant (LR) was PI-/Annexin V+, representing the early-stage apoptotic cells. (TIF) [file pone.0150954.s002.tif]
